# Patho-Physiology of Aging and Immune-Senescence: Possible Correlates With Comorbidity and Mortality in Middle-Aged and Old COVID-19 Patients

**DOI:** 10.3389/fragi.2021.748591

**Published:** 2021-12-16

**Authors:** Saba Farheen, Sudhanshu Agrawal, Swaleha Zubair, Anshu Agrawal, Fauzia Jamal, Ishrat Altaf, Abu Kashif Anwar, Syed Mohammad Umair, Mohammad Owais

**Affiliations:** ^1^ Interdisciplinary Biotechnology Unit, Faculty of Life Sciences, Aligarh Muslim University, Aligarh, India; ^2^ Division of Basic and Clinical Immunology, Department of Medicine, University of California, Irvine, Irvine, CA, United States; ^3^ Department of Computer Science, Aligarh Muslim University, Aligarh, India; ^4^ Department of Anatomy, HSZH Gov, Unani Medical College, Bhopal, India; ^5^ Ajmal Khan Tibbiya College, Aligarh Muslim University, Aligarh, India

**Keywords:** immunosenescence, inflammaging, comorbidities, SARS-CoV-2, COVID-19, innate and adaptive immune system

## Abstract

During the last 2 years, the entire world has been severely devastated by the severe acute respiratory syndrome coronavirus 2 (SARS-CoV-2) pandemic (COVID-19) as it resulted in several million deaths across the globe. While the virus infects people indiscriminately, the casualty risk is higher mainly in old, and middle-aged COVID-19 patients. The incidences of COVID-19 associated co-morbidity and mortality have a great deal of correlation with the weakened and malfunctioning immune systems of elderly people. Presumably, due to the physiological changes associated with aging and because of possible comorbidities such as diabetes, hypertension, obesity, cardiovascular, and lung diseases, which are more common in elderly people, may be considered as the reason making the elderly vulnerable to the infection on one hand, and COVID-19 associated complications on the other. The accretion of senescent immune cells not only contributes to the deterioration of host defense, but also results in elevated inflammatory phenotype persuaded immune dysfunction. In the present review, we envisage to correlate functioning of the immune defense of older COVID-19 patients with secondary/super infection, increased susceptibility or aggravation against already existing cancer, infectious, autoimmune, and other chronic inflammatory diseases. Moreover, we have discussed how age-linked modulations in the immune system affect therapeutic response against administered drugs as well as immunological response to various prophylactic measures including vaccination in the elderly host. The present review also provides an insight into the intricate pathophysiology of the aging and the overall immune response of the host to SARS-CoV-2 infection. A better understanding of age-related immune dysfunction is likely to help us in the development of targeted preemptive strategies for deadly COVID-19 in elderly patients.

## Introduction

The pandemic outbreak of a novel coronavirus–severe acute respiratory syndrome coronavirus-2 (SARS-CoV-2) has tumulted the whole world population into chaos since the first quarter of the year 2020. Over the course of subsequent few months, millions of coronavirus disease-19 (COVID-19) cases had been registered across the world (Mok et al., 2020). The SARS-CoV-2 infection has manifested a wide range of clinical consequences. In spite of the fact that the majority of infected people remain either asymptomatic (estimated to be between 17.9 to 78%) or have moderate disease, around ∼20% of the COVID-19 patients contract serious illness, with life-threatening pneumonia and acute respiratory distress syndrome (ARDS) (Yang X et al., 2020; Yin et al., 2021). A substantial subpopulation of COVID-19 patients exhibits systemic symptoms like secondary sepsis, cardiovascular and cardiac complexities, thrombo-embolism, coagulopathy, and as well as multi-organ malfunction (Huang et al., 2020; Yang R et al., 2020). Age is a significant risk factor for severe SARS-CoV-2 infestation. COVID-19 patients who are older or have pre-existing comorbidities are at higher risk of mortality (Elezkurtaj et al., 2021). In addition, elderly patients are more likely to have chronic comorbidities like diabetes mellitus, hypertension, cancer, obesity, and cardiovascular disorders, etc; all of which make them more susceptible to COVID-19 as well as associated complications (Huang et al., 2020; Zhang L et al., 2020; Zheng et al., 2020; Zhang X et al., 2020; Zhou F et al., 2020). The above specified comorbidities, however, are inadequate to explain why age is a risk factor in relenting to COVID-19, in its own right. Besides the elevated risk of older people succumbing to SARS-CoV-2 infection, there are several studies that suggest a great degree of difference in the sickness outcome between younger and older SARS-CoV-2 patients (Yang R et al., 2020; Zhang X et al., 2020; Lennon et al., 2020; Jung et al., 2020; Liu Yet al., 2020). In fact, despite comparable viral loads, younger COVID-19 patients are likely to be asymptomatic than older patients, according to a cross-sectional analysis of residents, and employees in nursing homes and assisted living facilities (Lennon et al., 2020). In Shanghai, a systematic analysis of clinical, genetic, and immunological data from 326 confirmed COVID-19 cases revealed that, among other factors, age was substantially associated with poor clinical outcomes (Zhang X et al., 2020).

The enveloped SARS- CoV-2 possesses a large (27.9–31 kb) positive sense single-stranded RNA as its core genome. The virus enters the human host generally via the respiratory tract. Once inside the host, the contagion employs its spike (S) protein to recognize the angiotensin-converting enzyme 2 (ACE2) receptor on the alveolar epithelial type II pneumocytes in the lung (Zhang H et al., 2020). Next, TMPRSS2 (host transmembrane protease serine 2) facilitates S protein activation and viral entry into the cell (Hoffmann et al., 2020). Inside the cell, it multiplies, and eventually the cell burst causes the release of new virus particles that infect adjacent cells, and also wander in the extracellular spaces to find susceptible cell targets in the host. Besides the lung, the ACE2 receptor is profusely expressed on epithelial cells of the gastrointestinal tract, kidneys, vascular endothelial cells, and adipose tissues, etc (Hamming et al., 2004; Al Heialy et al., 2020; Al-Benna, 2020; Albini et al., 2020; Gu et al., 2020; Li B et al., 2020; Thum, 2020). The expression of ACE2 receptors has also been reported on the gingiva, salivary gland, and taste cells of the oral cavity (Doyle et al., 2021; Huang et al., 2021). It is suggested that SARS-CoV-2 affects olfactory sensory neuronal cells indirectly, as the ACE2 receptor is not directly expressed on olfactory cells rather expressed on supporting cells (Brann et al., 2020). The stay of the SARS-CoV-2 inside the host causes progressing tissue damage that ensues in various related clinical signs and symptoms. The vast array, or distribution of the susceptible target cells in the inflicted host, may lead to varying degrees of clinical presentation as well as indications, involving respiratory symptoms like cough, shortness of breath, and fever being the most common. The digestive system associated symptoms like diarrhoea, nausea, and vomiting are also equally manifested. Thromboembolism and strokes have also been linked to clinical symptoms of vascular endothelial dysfunction and pathological coagulopathy-related manifestations (Oxley et al., 2020). Loss of taste and sense of smell are the commonly observed symptoms of COVID-19 (Brann et al., 2020; Huang et al., 2021).

The post COVID-19 complications may involve pneumonia and acute respiratory distress syndrome (ARDS), which require artificial ventilation and, if not managed appropriately, may be fatal. ARDS is clinically characterized by acute respiratory failure, hypoxemia, bilateral lung infiltrates on chest imaging that is not entirely explained by effusions, collapse, and/or nodules, and a lack of cardiogenic-related edema (Papazian et al., 2016). Patients with severe COVID-19 had been reported to have elevated levels of circulating cytokines and chemokines (cytokine storm), which increases their chance of developing pneumonia, ARDS, sepsis, and coagulopathy, some of the serious complications associated with COVID-19 (Huang et al., 2021; Iba et al., 2019). Alongwith ACE2 receptors, dipeptidyl peptidase 4 (DPP4) also known as CD26 may be a binding target for SARS-CoV-2. In fact, the CD26 molecule (DPP4) has been described as a binding receptor for MERS-CoV, another prominent member of the CoV family (Valencia et al., 2020). The protein docking studies, based on crystal structure, revealed the high affinity between human dipeptidyl peptidase 4 (DPP4), and the spike (S) receptor-binding domain of SARS-CoV-2 (Li et al., 2020a). The DPP4 expression is more common in senescent cells. This may form the basis for the high occurrence of COVID-19 in elderly population. Moreover, enhanced expression of DPP4 in the obese individuals or type 2 diabetic patients correlates with the higher proliferation of human smooth muscle cells and increased expression of pro-inflammatory cytokines like MCP-1, IL-6, and IL-8 via NF-ҡB activation leading to damaging consequences of SARS-CoV-2 in the inflicted patients (cf. older as well as diabetic patients) (Valencia et al., 2020). Hence, inhibitors of the DPP4 might prove as strategic therapeutic agents as they reduce the level of IL-6, which plays a key role in the inflammatory response in SARS-CoV-2 patients (Valencia et al., 2020; Solerte et al., 2020). Both the innate and the adaptive immune systems are highly involved in the regular immune response to viral infections, including coronaviruses. The innate immune response comprises toll-like receptors (TLR), which are membrane-bound pattern-recognition receptors (PRRs), expressed by dendritic cells (DC) and macrophages, recognize viral nucleic acids, which is followed by intracellular signaling and synthesis of antiviral type I interferon (IFN) in antiviral defense mechanisms (Kawasaki and Kawai, 2014). TLR7 and TLR8, which sense single-stranded RNA, and may be TLR3, which senses double-stranded RNA intermediates, and are expected to be the first receptors to detect SARS-CoV-2 in the host cells (Khanmohammadi et al., 2021). An interactive association between Myd88 and specific TLRs (TLR1, TLR2, TLR4, TLR5, TLR8, and TLR9) has been suggestively linked with disease progression in COVID-19 patients. In K18-ACE2 transgenic mice, TLR2 has been reported to play important role in sensing the SARS-CoV-2 envelope protein that eventually ensues in profuse inflammatory cytokine production (Zheng et al., 2021).

In general, natural killer (NK) cells play a significant role in imparting antiviral defense. Furthermore, the expression of proinflammatory cytokines such as IL-6, IL-1β, tumour necrosis factor (TNF-α), and chemokines by the active immune cells helps in accumulation of neutrophils and other inflammatory immune cells to the infection site (Bajaj et al., 2021). An adaptive immune response begins when viral antigen is presented to virus-specific CD4 helper T cells and CD8 cytotoxic T cells. Antigen-specific CD8 T cells are essential for destroying virally infected cells, whereas cytokines such as IFN-γ are important for the activation of antigen-presenting cells including macrophages (Bajaj et al., 2021). CD4 helper T (Th) cells are critical for supplying cognate assistance to B cells, which undergo class switching and somatic hypermutation to switch antibody synthesis in favor of IgG isotype, which is more specific, precise, and display a high affinity for neutralization (Bajaj et al., 2021). Various active immune cell populations, including neutrophils, and NK cells, have a great deal of potential to inhibit invading viruses in the circumstances when expression of MHC-I molecules had been downmodulated to a significant level (Bajaj et al., 2021). Markedly, the immune response is a two-edged sword: on the one hand, it mediates defensive immunity and is necessary; on the other hand, excessive and improper development of inflammatory cytokines may contribute to cytokine storm syndrome, which causes organ immunopathology and death of the infected host (Zhou Y et al., 2020).

Normal aging is followed by progressive biological alterations in various systems of the host. Some of these contribute to the weakening of the immune system’s functions (Bajaj et al., 2021). The elderly subjects are more susceptible as well as vulnerable to respiratory infections like influenza and novel coronaviruses. Age-related immune-inflammation, or inflammaging, and associated inflammatory diseases are more common in the elderly population (Franceschi et al., 2000; Shaw et al., 2013; Fulop et al., 2018). These alterations, in combination with comorbidities, make elderly people more prone to latent or new infections, resulting in elevated COVID-19 morbidity, and death.

A superior repertoire of naive immune cells in younger people endows them with the potential to fight new infections more profoundly. Young people can withstand pathogen onslaught more successfully. As a consequence, exposure to the virulent pathogen may result in milder or even asymptomatic infection (Bajaj et al., 2021). The majority of younger people who test positive for the novel coronavirus, however, do not show disease symptoms. Exposure to the whole pathogen or any of their antigen components may cause upregulation of MHC molecules, costimulatory surface structures on pathogen-specific T and B cells of healthy individuals. This results in a bigger and more robust pool of effector molecules and their precursors that help in the recognition of a broader spectrum of foreign entities and eventually defend against new infectious diseases such as the COVID-19 pandemic (Bajaj et al., 2021). Interestingly, several studies have demonstrated the effect of aging on host immune responses against viral infections. Immunosenescence, for example, relates to diminished vaccination effectiveness and increased vulnerability to infectious diseases in the elderly. Vaccines for infections like influenza and shingles have been in practice for a long time; though, the poor efficacy and efficiency in the elderly reflect an aging-related drop in vaccination-induced immunogenicity (Oh et al., 2019).

In this review, we will discuss the concept of immunosenescence and inflammaging, as the correlates associated with physiological changes in the immune system of the elderly population, and how they impact the host immunological response to viral infections, mainly SARS-CoV-2. Further, we will present an overview of current studies on senescence-induced dysregulation of the immune system, with an emphasis on functional analysis of innate and adaptive immune cells, as well as its potential influence on viral immune responses, and such as COVID-19.

## Immunosenescence and Inflammaging

**FIGURE 1:**
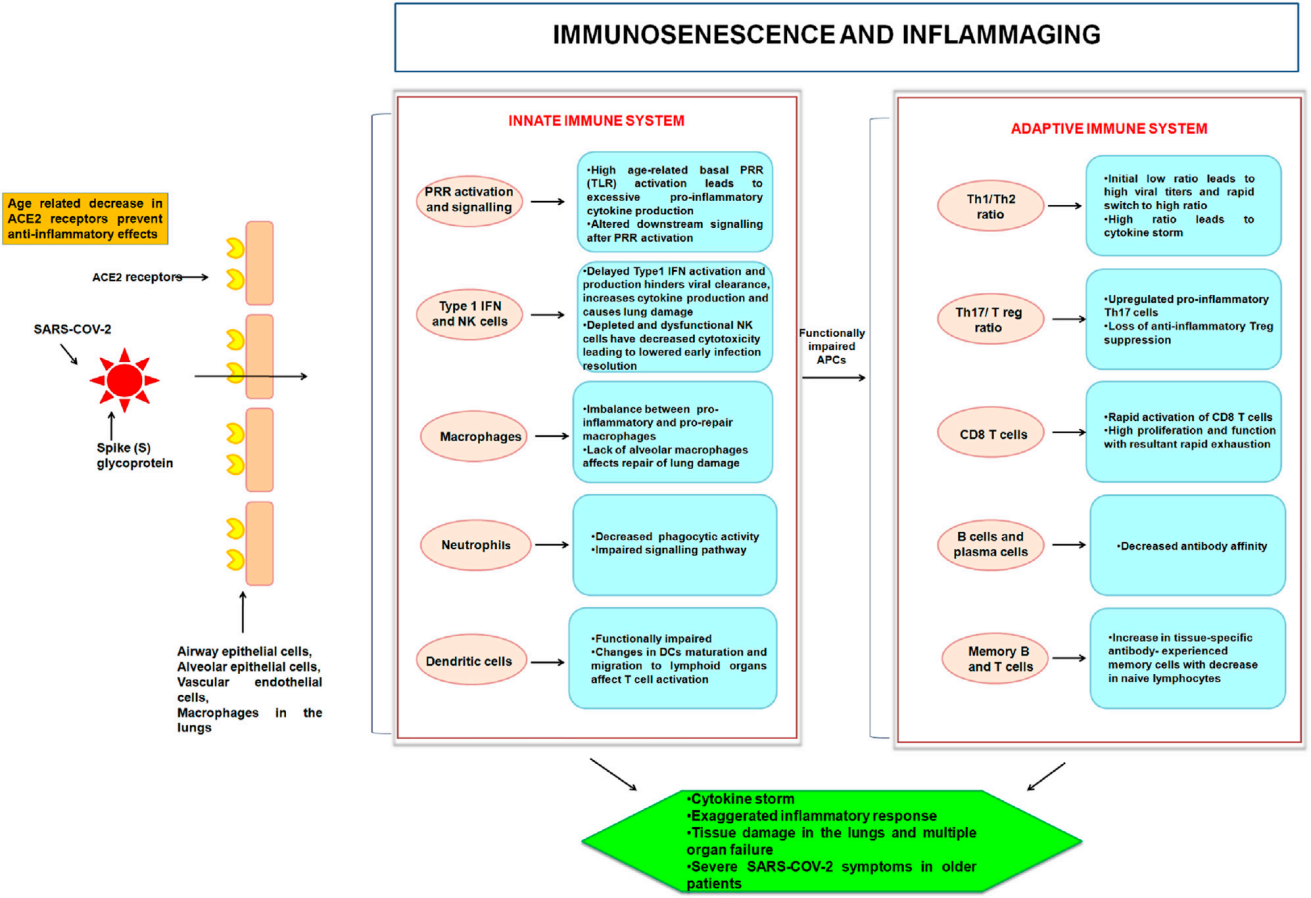
Age-linked modifications in the innate and adaptive immune systems with relevance to COVID-19 are depicted in this illustration. SARS-CoV-2 mediated infliction of parenchymal cells (through ACE2 receptor) ensues in activation of PRR of the innate immune system of the host. With age, cellular activities and signaling become deranged. This hampers the innate activation of an already diminished adaptive immune system. The abnormalities result in “inflammaging” from proinflammatory innate immune responses and adaptive immunological immunosenescence.

Immunosenescence is a term used to describe the degeneration of the immune system. An age-related change, in both the innate and adaptive immune systems, contributes to the development of a chronic inflammatory state, and thereby results in a decreased ability to combat against the new infection (Stahl and Brown, 2015) (Figure 1). The deterioration in the immune robustness makes the host susceptible to infectious diseases. This may also result in the inadequate immune response to vaccination or increased susceptibility to cancer and autoimmune diseases. Immunosenescence reflects a relatively complex shift in the activity across many immunological responses, rather than just a loss of immune cells in terms of number. Because of a decline in peripheral naive T and B cells, the immune system’s capacity to respond effectively to novel antigens is weakened (Stahl and Brown, 2015). Immunosenescence is also linked to the accumulation of memory T cells (Aiello et al., 2019). Antigen load over time, alongwith reduced lymphopoiesis, causes a decline in naive T cell populations and accretion of memory cell subsets (Aiello et al., 2019). Aging also comprises the loss of expression of the co-stimulatory surface protein CD28 from both CD4 and CD8 T cell populations. The less copy number of costimulatory CD28 molecule is a major hallmark of senescent T cells (Weyand and Goronzy, 2016). Loss of expression of CD27, another costimulatory molecule, and has also been associated with T cell senescence (Larbi and Fulop, 2014). The loss of CD28 and/or CD27 co-stimulatory molecules in T cells can be recognized as a sign of short telomeres and reduced telomerase activity, an indicator of replicative senescence (Larbi and Fulop, 2014). A variety of stress and aging-related illnesses are linked to reduced telomere length in leukocytes (de Punder et al., 2019). The expression of CD57 and KLRG-1 (killer-cell lectin like receptor G1, a co-inhibitory receptor) on T-cells is the marker of senescent T cells, as they are considered to be inhibitors of proliferation, shows replicative senescence (Larbi and Fulop, 2014). CD57^+^ cells have short telomeres, poor telomerase activity, reduced expression of cell-cycle genes, and restricted proliferative potential (Focosi et al., 2010). Senescent T cells persist and accumulate during aging, and secrete high levels of proinflammatory cytokines, chemokines, growth factors, and proteases. These factors represent a typical hallmark of senescence and therefore they have been defined as senescence-associated secretory phenotype (SASP) (Bartleson et al., 2021). Senescent cells frequently modify their gene expression, rendering themselves less prone to apoptosis (Alves and Bueno, 2019).

An imbalance of regulatory and stimulatory mediators, such as cytokines and acute phase reactants, also exemplifies the immune system’s aging. This leads to a sub-clinical chronic proinflammatory condition known as inflammaging. The age-related rise in the number of senescent cells, together with the immune system’s reduced capacity to eliminate these cells, creates an environment rich in pro-inflammatory cytokines and reactive oxygen species (ROS), which pushes surrounding cells toward senescence (Stahl and Brown, 2015). Chronic activation of the innate immune system and increased release of inflammatory mediators may have a direct correlation with inflammaging that is generally accompanied by increased proinflammatory NF-κβ activation (Salminen et al., 2018). The unregulated inflammatory response to SARS-CoV-2 results in both local and systemic tissue damage. Most of the patients with severe COVID-19 have significantly increased levels of pro-inflammatory cytokines and chemokines in the serum, including IL-6, IL-2, IL-1β, IL-8, IL-17, granulocyte (G)-colony-stimulating factor (CSF), granulocyte-macrophage (GM)-CSF, C–X–C motif chemokine ligand (CXCL)10, C–C motif chemokine ligand (CCL)2, CCL3, and TNF (Xu et al., 2020; Qin et al., 2020). In a study involving aging mice, it was found that similar cytokines, such as IL-6, IL-1α, IL-1β, TNF-α, and the chemokine CCL2 were also associatedwith the aged host response to SARS-CoV-2 (Dinnon et al., 2020; Leist et al., 2020). Inflammaging causes a rise in several of these cytokines in the elderly. For example, increased level of IL-6 has been considered as a predictor of poor outcomes in COVID-19 patients, because of the fact that IL-6 is indeed one of the most accurate aging indicators (Johnson, 2006). The expression of IL-6 cytokine is a clinical sign of NF-κB (a transcription factor) activation in the vascular system. NF-κB regulates many pro-inflammatory genes of innate immune cells, in fact, there is a great deal of correlation between aging, and NF-κB signaling and inflammation (Salminen et al., 2008; Brasier, 2010).

## Remodelling of the Innate Immune System With Aging

### Toll-like Receptors

The innate immune system uses germline-encoded pattern-recognition receptors (PRRs) in the initial identification of microbes (Kawasaki and Kawai, 2014). PRRs are mainly expressed by dendritic cells, macrophages, neutrophils, monocytes, and epithelial cells. They perceive microbe-specific molecular signatures known as pathogen-associated molecular patterns (PAMPs) as well as self-derived molecules derived from damaged cells known as damage-associated molecular patterns (DAMPs) (Kawasaki and Kawai, 2014). Toll-like receptors (TLRs) are the membrane-bound PRRs that are highly conserved, capable of detecting a wide range of stimuli such as viral DNA/RNA, bacterial lipopolysaccharides, lipoproteins, and nucleic acid, *etc*; which serve as PAMPs (Kawasaki and Kawai, 2014). TLRs are important regulators of innate immunity against microbial infections in the innate immune system. Recent studies have added to the current insights in animal models by elucidating the effects of aging on TLR function in human cohorts. With increasing age, TLR expression and activity in monocytes (Van Duin et al., 2007), DCs (Panda et al., 2010), and neutrophils (Montgomery and Shaw, 2015) decreases. The splenic and peritoneal macrophages of elderly female C57BL/6 mice (18–24 months old), as well as older humans (≥65 years), had been reported to possess lower levels of TLR expression (Renshaw et al., 2002). While most TLRs are upregulated upon interaction with specific ligands in the macrophages of the young, the TLR expression, in the macrophages of elderly mice, is weakly upregulated, and in particular, expression of TLR3 is poorly identified (Renshaw et al., 2002).

Surprisingly, not all different types of TLRs were found to be downregulated in the elderly subjects. TLR1 surface expression, for example, declines with age, and but TLR2 surface expression remains constant (Van Duin et al., 2007). Low TLR1 surface expression and impairment in TLR1/2 signaling in the elderly resulted in reduced MAPK signaling, which in turn reduces the production of TNF-α and IL-6 in the host monocytes. After activation with TLR ligands, such as TLR1/2, TLR2/6, TLR3, TLR4, TLR5, and TLR9 agonists, macrophages from aged mice produce reduced amounts of IL-6 and TNF-α (Renshaw et al., 2002).

In contrast, upon interaction with specific ligands, monocytes from elderly human beings had upregulated expression of TLR5 molecule. This in turn resulted in enhanced production of TLR5-induced cytokines (Qian et al., 2012). TLR5 signaling is effectively maintained during aging, according to Lim et al., who reported that *in vitro* overexpression of caveolin-1 increased TLR5 mRNA via the MAPK pathway and prolonged TLR5 half-life via direct contact (Lim et al., 2015). Principally, TLR expression declines with age, with the exception of TLR5. TLR mislocalization can lead to changes in the expression of cytokine and chemokine thereby affecting the functioning of the immune system of the host (Qian et al., 2012).

TLR-7 and TLR-8, expressed in the endosomal compartments of respiratory epithelial cells, are the two TLRs that play a key role in sensing ssRNA of invading SARS-CoV-2 (Moreno-Eutimio et al., 2020; Onofrio et al., 2020; Sallenave and Guillot, 2020). The production of both IFN regulated cytokines, as well as pro-inflammatory cytokines, were induced by TLR7, and TLR8 respectively (Moreno-Eutimio et al., 2020; Onofrio et al., 2020). Aging impairs the migration of innate immune cells and signaling events following PRRs activation resulting in increased production and dysregulation of cytokines, commonly known as cytokine storm (Shaw et al., 2013; Onofrio et al., 2020). Innate immune cells with age-related changes in TLR expression can still produce cytokines. In human monocytes, for example, reduced TLR1 surface expression combined with lower TLR1/TLR2 might cause cytokine production. The observed post-translational aberration has been reported to worsen with age (Shaw et al., 2013; Onofrio et al., 2020). The production of a plethora of cytokines in the host, post SARS-CoV-2 infestation implies, high basal TLR activation, which cannot be further stimulated in response to a pathogen origin ligand, and may result in innate immune system failure (Shaw et al., 2013; Onofrio et al., 2020).

### Monocytes and Macrophages

Even though there are no significant variations in the number of total monocyte subsets between the young (21–40 years) and the elderly (≥65 years) subjects, a worldwide study of circulating monocytes in various age groups reveals substantial age-related alterations (Metcalf et al., 2015). Non-classical CD14^+^CD16^+^ monocyte counts, for example, increased dramatically with age, however, exhibited underexpression of HLA-DR, and chemokine receptor CX (3)CR1 in the elderly. On the other hand, classical CD14^+^CD16^−^ monocyte numbers, did not change with age, despite the fact that serum MCP-1 levels elevate with age, but not MIP-1α, MIP-1β, and or fractalkine (CX3CL1) (Seidler et al., 2010). Human monocyte subsets responded differently to TLR agonists depending on their age, resulting in changes in surface molecule expression and lower production of interferons, and cytokines such as IL-1β (Metcalf et al., 2017). Monocytes from elderly people show reduced phagocytosis yet have shorter telomeres and considerably greater intracellular levels of TNF-α both at the basal level and after TLR4 activation, suggesting that the elderly have dysfunctional monocytes (Hearps et al., 2012).

In general, the functioning of macrophages is impaired by aging, along with the reduction in monocytic activity. TLR expression on macrophages is decreased in elderly humans and mice, as previously documented (Renshaw et al., 2002; Shaw et al., 2011). Macrophages are significantly prevalent as a pro-inflammatory M1 phenotype in healthy old adipose and hepatic tissue, and as an immunosuppressive M2 phenotype in old lymphoid tissues, lung, and muscle (Jackman et al., 2017). In aged people, the buildup of immunological complexes, cytokines, hormones, free fatty acids, oxidized low-density lipoproteins, and immunoglobulins readily activate macrophages (Acharya et al., 2020). This generally ensues in low-grade inflammation, which in turn compromises the overall efficacy of the immune response (Acharya et al., 2020). The expression of IFN family cytokines results in the upregulation of C- reactive protein in the infected host. Due to decreased IFN production, there is an imbalance between proinflammatory, and pro-repair airway macrophages in patients with SARS-CoV-2 (Acharya et al., 2020). Chelvarajan *et. al*. described decreased TNF-α and IL-6 production and enhanced IL-10 production in elderly (22–24 months old) BALB/c and C57BL/6 mice after activation with TLR ligands (Chelvarajan et al., 2005). Also, the CD14 and TLR4-expressing cells had been reported to downfall resulting in a decrease in cytokines including IL-6, TNF-α, IL-1β, and IL-12 in the host (Chelvarajan et al., 2005). Inflammaging can lead to the buildup of alternatively activated (M2-like) macrophages in tissues, which remain pro-inflammatory, and exhibit senescence markers. It can be inferred that aging affects a variety of activities in macrophages, including TLR signaling, polarisation, phagocytosis, and wound healing, *etc*. (Van Beek et al., 2019).

### Dendritic Cells (DCs)

Several studies have found that while the numbers of myeloid DCs (mDCs) do not change with age, the number and function of plasmacytoid DCs do decline (Shodell and Siegal, 2002; Della Bella et al., 2007; Jing et al., 2009). It is worth noting that the number of specialized DCs in the epidermis and mucosal tissues, such as Langerhans cells, decreases as people get older (Schwartz et al., 1983; Sprecher et al., 1990). DCs from the aged people have considerably decreased capacity to phagocytose antigens on a functional level (Pietschmann et al., 2000). When exposed to LPS, ssRNA, or self-DNA, aged mDCs produce more IL-6 and TNF-α than young mDCs. This induction had been correlated to age-related changes in signaling pathways that lead to the altered PI3K, NF-ҡB, or type I IFN responses. DCs function can be impacted by age-related alterations in signaling pathways, which can result in defective cytokine production in response to pathogens or self-DNA, as well as decreased phagocytosis, and migratory capacities (Agrawal et al., 2007; Agrawal et al., 2009). In SARS-CoV-2 patients, changes in the lung microenvironment also impact DCs maturation and migration to lymphoid organs, influencing T cell activation (Tay et al., 2020).

### Neutrophils

Neutrophils, major phagocytic cells that specialize in imparting protection against invading pathogens in the early stages of infection (Hong, 2017). While the neutrophil cell count does not vary as people get older, their phagocytic and chemotactic capacities do (Wenisch et al., 2000). Nevertheless, the recruitment of neutrophils to the site of infection is affected by aging, as evidenced by studies that have found decreased neutrophil-mediated chemotaxis in aged hosts (Niwa et al., 1989; Wenisch et al., 2000). Neutrophils require a high level of energy to carry out phagocytosis, aging downgrades hexose transport and raises intracellular calcium levels, preventing the uptake of energy, and as a result, phagocytosis. Age-linked abnormalities in neutrophils are mostly caused by defective signal transduction pathways such as poor anti-apoptotic responses regulated by the JAK-STAT tyrosine kinase and PI3K-AKT pathways (Shaw et al., 2013; Jackman et al., 2017). The phosphorylated form of the PI3K regulates phagocytosis, degranulation, and chemotaxis of the neutrophil population. In the elderly population, the elevated activity of PI3K causes neutrophils to migrate imperfectly and secrete proteases in the surroundings that harm normal tissue rather than aberrant tissue at the site of inflammation or infection (Sapey et al., 2014). It has been demonstrated that like other viruses, SARS-CoV-2 can also stimulate neutrophil extracellular traps (NETs) in a process called NETosis in human neutrophils (Arcanjo et al., 2020). However, aged neutrophils generate reduced neutrophil extracellular traps (NET), which are consisted of nuclear components and granule proteins, and are capable of binding and trapping extracellular pathogens to protect the body from infection (Tseng et al., 2012; Hazeldine et al., 2014). As a result, a decrease in NET production with age might cause delayed wound healing and a greater vulnerability to invading pathogens (Tseng et al., 2012).

### Type 1 IFN and NK Cells

PAMPs and DAMPs activate PRRs, which trigger a signaling cascade that results in the production of type I IFN and other inflammatory mediators during a pathogenic invasion (Acharya et al., 2020). During the antiviral defense, early activation and generation of type I interferon are beneficial. Type I IFN inhibits viral replication and dissemination by acting as an early antiviral response through autocrine and paracrine type I IFN receptor (IFNAR) signaling (Nikolich-Žugich, 2018; Acharya et al., 2020). Delayed production of IFN might cause tissue injury and inflammatory cytokine storm. The time-lapse in the production of type I IFN increases with age and has also been observed in the SARS-CoV-2 patients in general (Nikolich-Zugich et al., 2020; Acharya et al., 2020). Furthermore, the SARS-CoV-2 virus adapts immune evasion mechanisms to preclude the early production of type I IFN. It has been reported that SARS-CoV-2 type I IFN is very less in individuals with serious SARS-CoV-2 symptoms, in addition to delayed local IFN responses (Aiello et al., 2019; Acharya et al., 2020). The altered production of IFN led to an unbalanced state between proinflammatory macrophages and reparative airway macrophages (Aiello et al., 2019; Acharya et al., 2020). Host susceptibility to severe COVID-19 disease can be increased by either genetic or acquired abnormalities in type I interferon signaling. Bastard *et al.* employed sensitive immunoassays and neutralization assays to determine the existence of autoantibodies against α, β, and ω type I interferons in plasma samples from a large cohort of COVID-19 patients and uninfected controls (Bastard et al., 2021). Furthermore, it has been discovered that nearly 10% of patients with severe COVID-19 pneumonia had significant titers of neutralizing autoantibodies against type I IFN-2α and IFN-ω (Bastard et al., 2020). Infected patients, who were asymptomatic or had milder symptoms, as well as healthy people, did not have these autoantibodies. In the control group, the incidence of neutralizing autoantibodies to type I interferon increased with age, peaking at the age of 70. These data suggest that autoantibodies against type I IFNs are a prevalent form of acquired immunodeficiency that accounts for around 20% of all COVID-19 deaths (Bastard et al., 2021). During coronavirus infections, IFN modulates other immune cells such as NK cells, and which are not only down numbered rather become dysfunctional in individuals with advanced-stage SARS-CoV-2 infection (Aiello et al., 2019; Acharya et al., 2020). In elderly people, alteration in the activity of NK cells is linked to increased severity of infection that is accompanied by death (Shaw et al., 2013). There are reports which suggest that type I IFN downmodulates NK cell functioning in elderly mice (Plett and Murasko, 2000). Furthermore, in response to the virus, the synthesis of IFN by plasmacytoid DCs diminishes as people get older (Agrawal, 2013). As a result, the reduced and delayed production of type I IFN, as well as the altered production of NK cells in elderly people, affect the first-line antiviral response to SARS-CoV-2 invasion, and reduce the possibilities of infection resolution at an earlier stage than in younger people (Shaw et al., 2013; Aiello et al., 2019; Acharya et al., 2020).

## Comorbidities and Innate Immune Response

SARS-CoV-2 symptoms are more evident in older patients (>60 years) and individuals with a history of comorbidities, owing to hyperactive chronic innate inflammatory responses as well as structural and functional alterations in organs (Frasca et al., 2017). Obesity boosts the difficulties linked with the virus and aging, in part, owing to structural/functional abnormalities and also due to visceral fat that can act as a viral repository and exacerbate inflammatory responses (Iacobellis, 2020; Malavazos et al., 2020; Ryan and Caplice, 2020). In both older (>60 years) as well as younger (under the age of 40) COVID-19 patients with severe disease compared to those with mild disease, Computed Tomography (CT) imaging revealed increased fatty liver and epicardial adipose tissues, implying obesity as a potential predictor of COVID-19 severity (Iacobellis et al., 2020b; Deng et al., 2020). Additionally, adipose tissues function as endocrine organs, producing hormones such as leptin and adiponectin, as well as inflammatory cytokines like IL-6 and TNF (Banerjee et al., 2020). Since obese and diabetic patients have more adipose tissue, they have more ACE2 expressing adipocytes, thus increasing their susceptibility to viral invasion in the adipose tissue, which might lead to greater severity of SARS-CoV-2 infection (Banerjee et al., 2020; Fajnzylber et al., 2020). In obese people, innate immune cells like NK cells, macrophages, and neutrophils, as well as adaptive immune cells, enter the adipose tissue and trigger inflammatory responses through the release of pro-inflammatory cytokines, metabolic alterations, and phenotypic and functional abnormalities (Frasca et al., 2017). Obesity is also a potential risk factor for endothelial dysfunction, which, when coupled with SARS-CoV-2 caused endothelial cell damage, ensuing in increased endothelial dysfunction that eventually worsens associated problems (Engin, 2017; Amraei and Rahimi, 2020). The combination of age-related defective patterns in many components of the innate immune inflammatory responses prevents the virus elimination from the host (Frasca et al., 2017). It explicitly implies that persons over the age of 60 and those with co-morbidities are at a greater risk of SARS-CoV-2 severity because they are more susceptible to induce a defective immune response and have relatively high rates of morbidity and mortality.

## Modulation of the Adaptive Immune System With Age

### B Cells

B lymphocytes are key regulators of the adaptive immune system, and play role in various processes including antibody synthesis to cytokine release (Ponnappan and Ponnappan, 2011; Nikolich-Zugich et al., 2020). Invading pathogens as a strategy to counter host immune system onslaught on one hand and host-related other factors (comorbidities and age-related) may deform the functioning of the host B cells (Ponnappan and Ponnappan, 2011; Nikolich-Zugich et al., 2020). Deformity in the quality of secreted antibodies has been investigated more extensively in the context of aging than any of these two roles (Ponnappan and Ponnappan, 2011; Nikolich-Zugich et al., 2020). Since B cells must produce a variety of specific antibodies to identify a wide range of threatening antigens, heterogeneity is important in the B cell repository for an effective immune response (Anolik et al., 2009). Aged people often have a constrained B cell repository, which could make them more susceptible to infectious diseases, less able to react to vaccinations, and more likely to produce autoreactive Abs (Ponnappan and Ponnappan, 2011). According to a previous study, aging can affect the selection procedure during B cell affinity maturation (Banerjee et al., 2002), and as people get older, the B cell repertory becomes less diversified, with indications of non-pathogenic clonal expansions (Gibson et al., 2009). Poor vaccination responses against various infectious diseases are believed to be linked to the host immune system that loses its potential to generate differentiated antibody pools with expanded and diverse recognition (Goronzy and Weyand, 2013). Aging lowers the number of naive B cells and plasma cells whereas increasing the numbers of CD27^+^ memory B cells in mice (Fecteau et al., 2006). Though B cell lymphopoiesis is active throughout life, there is a reduction in B cell synthesis in elderly populations as bone marrow is degraded, and also the percentages and quantities of human peripheral B cells significantly reduced with age (Zharhary, 1988; Rossi et al., 2003; Frasca et al., 2008).

Alterations in the B cell population can be linked to Ab production, in which B cells reduce their capacity to generate an adequate Ab response against naive as well as known antigens, as they get older. Furthermore, in the aged host, the Ab response is accompanied by poor isotype switching and a brief time of activation (Nikolich-Žugich, 2018). Also, reduced affinity is not just related to lowered production of B cells, rather related to decreased rates of isotype switching, and less somatic hypermutation in the aged immune system (Henry et al., 2019). The class switch recombination and somatic hypermutation are the two cellular mechanisms that produce newer classes of Abs. The process is mainly regulated by activation-induced cytidine deaminase (AID). In older people, impairment in MAPK signaling as well as the reduction in mRNA stability and DNA binding affinity of transcriptional factor E47 (encoded by E2A), ensues in the downregulated expression of AID gene (Oh et al., 2019). As a result of these internal changes in B cells, Ab diversity is eventually reduced. The microRNA of the B lymphocytes such as miRNA-155 and miRNA-16 had been described as major contributors to the molecular changes linked with B cell senescence mediated by the downregulation of AID and E47 (Frasca et al., 2015). Downregulation of E2A caused by aging promotes abnormalities in class switch recombination of IgM memory B cells, and reduced expression of AID gene in B cells has been found to cause downmodulation of class switch recombination in the elderly subjects (Van der Put et al., 2004; Frasca et al., 2004). Thus, reduction in the expression levels of AID and E47 in the elderly population is expected to diminish the size and the number of germinal centers, in which mechanisms of Ab affinity maturation, including somatic hypermutation take place, therefore, decrease Ab affinity maturation, and the frequency of circulating Abs from plasma cells (Frasca, 2018).

The levels of IgG antibody specific for viral spike (S) protein in rhesus macaques are lower in elder macaques compared to younger ones during the early stages of acute SARS-CoV-2 infection which indicates a strong correlation of aging with impeded production of class-switched antibody titers required to eliminate the viral infection (Singh et al., 2021). The upregulation of age-linked attributes or features has been reported in B cells of aging animals as well as human beings (Cancro, 2020). TLR7 involvement is also important, considering the fact that it is a significant PRR for SARS CoV-2 mediated stimulation of host B cells (Cancro, 2020). These cells, populated in the late memory fraction (IgD^−^ CD27^−^, also known as double-negative B cells), release inflammatory molecules like TNF-α, IL-8, and IL-6, have been linked to autoimmune illness, chronic viral disease, and COVID-19 (Frasca et al., 2017; Cancro, 2020).

### T Cells

The T lymphocytes play a major role in the containment of virus-based infections. The role of T cells during aging is being emphasized due to their impact on overall immunological responses. Some of the impacts of aging include a gradual drop in the formation of freshly naive T cells, a more limited TCR repertoire, and ineffective activation of T cells (Salam et al., 2013). T cell output drops by 90% at puberty due to the involution of the thymus, which transforms into adipose tissue. Around the age of 40–50, T cells production declines again, and the remainder of the thymus degenerates (Nikolich-Zugich et al., 2020). The generation of naive T lymphocytes is reduced to just 1% of what it was before primary involution due to secondary atrophy. Furthermore, as people become older, lymph nodes become less capable of storing, and maintaining naive T cells (Nikolich-Zugich et al., 2020). Except in the extremely old, overall T cell counts do not drop with age, and this is owing to a compensatory increase in peripheral T cell proliferation, known as homeostatic proliferation, which is followed by the procurement of activated/memory cell phenotypes (Sprent and Surh, 2011; Ponnappan and Ponnappan, 2011). This indicates that, whereas the elderly have similar lymphocyte counts to younger people, their capacity to develop an adaptive response to a novel antigen is considerably reduced as a result of the exchange of naive T cells with antigen-independent experienced cells (Ponnappan and Ponnappan, 2011). Insignificant counts of naive T cells were linked to severe outcomes of COVID-19 (Moderbacher et al., 2020). This study might point to a link between the reduced naive TCR repertoire of the elderly before SARS-CoV-2 infection and poor disease outcomes.

Aging causes complex changes in T cells, affecting a variety of T-cell subsets such as naive, memory, and effector T-cells, and T_EMRA_ cells (Dixit, 2012). However, IL-7, a critical maintenance component of T cell homeostasis, does not vary with age, it usually reduces the number of naive T cells, and increases the number of senescent T cells (Nikolich-Žugich, 2018). Elderly T cells go through specific alterations that restrict antigenic specificity and decrease TCR expression, resulting in an age-related shift in gene expression which is induced by TCR in human CD4^+^ T cells (Bektas et al., 2013). TCR diversity was shown to be reduced with age utilizing the high-throughput illumina sequencing technology, revealing a substantial drop in the number of naive T cells, and TCR-beta diversity by the age of 40 (Tseng et al., 2012). Furthermore, thymus involution and changes in transcriptional factor expression levels result in dysfunctional T cells, which cause inflammaging and a greater risk for infection by lowering vaccination effectiveness (Britanova et al., 2014).

The rise of poor T cell activity over time was linked partly due to the age-related decrease of immunity or immunosenescene. Rather than becoming inefficient, senescent T cells, particularly those in the CD8 subpopulation, lose major elements of their TCR signaling machinery while gaining Natural Killer (NK cell) features. This suggests that T cells gain a new functional viewpoint when they develop towards a proliferative end-stage, irrespective of their antigen specificity (Abbas and Akbar, 2021). As cytokines are important regulators of the immunological response mediated by T cells, it has been suggested that altered cytokine production may cause age-linked defects in T cells. It has been found that elderly T cells mainly have a Th2-like phenotype and indicated by a shift in cytokine profiling (Mansfield et al., 2012). Th17 cells protect the body against extracellular pathogens and have been linked to the onset of autoimmune and chronic inflammatory disorders in individuals (Sandquist and Kolls, 2018). In the elderly, the ratio between Th17 and Treg seems to rise and it has been speculated that this change in the ratio might explain why the elderly are more prone to autoimmune diseases and have weakened immune responses to pathogens (Schmitt et al., 2013).

**FIGURE 2:**
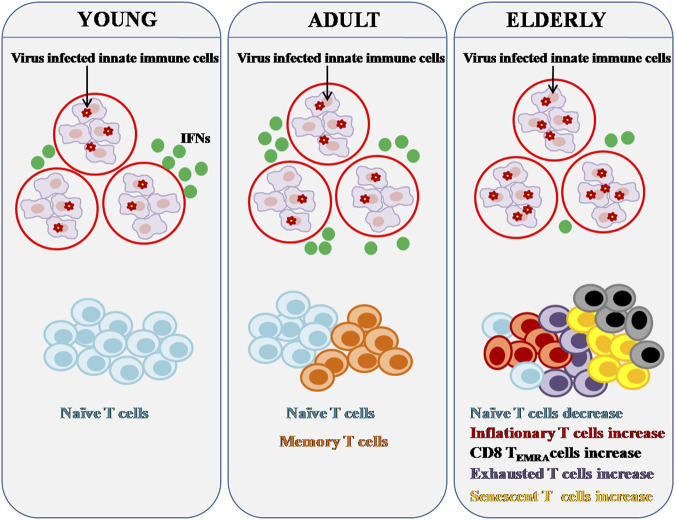
Effect of Immunosenescence on chronic viral infection and immunity. Major components of both the innate and adaptive immune systems alter as people age. Innate immune cells can activate IFN pathways in response to viral infection in order to clear virus-infected cells. Reduced IFN production can be caused by age-related abnormalities in innate immune cells. Persistent viral infection can lead to significant changes in adaptive immunity, particularly T cell composition and function. There are fewer naive T cells in the aged, but there are more senescent, inflationary, and or exhausted T cells that are functionally inert or dormant.

The influence of immunosenescence on T cells engaged in persistent viral infections is seen in Figure 2. Aging causes a drop in naive CD8^+^ T cells, a decrease in the heterogeneity of TCR repertoire, and an increase in senescent, exhausted, terminally differentiated T_EMRA,_ and inflationary T cells. It has been described that PD-1^+^/CD153^+^ memory phenotype CD4^+^ T cells as the senescence-associated T (SA-T) cell phenotype (Tahir et al., 2015). Both the TCR-mediated proliferation as well as T cell cytokine generation are impaired in SA-T cells, indicating cellular senescence. These cells release pro-inflammatory cytokines, which can build up and cause chronic inflammation in tissues (Fukushima et al., 2018).

Along with a rise in senescent T cells, the elderly experience a gradual and sustained growth of inflationary T cells (Kim et al., 2015). TCR repertoire is restricted in inflationary T cells, and so are the lymph node homing signals that cause accumulation in non-lymphoid peripheral organs and co-stimulatory receptors, as well as large amounts of inhibitory receptors (Snyder et al., 2008). Memory T cells produced by the elderly population may be poorly maintained in the peripheral repertoire, possibly due to niche competition caused by memory inflation (Klenerman and Oxenius, 2016). COVID-19 severity has been connected to the prevalence of cytomegalovirus (CMV), a major pathogen related to memory inflation, but additional research is required to determine how CMV, as well as other latent viruses, influence the production of new memory T cells, function, and longevity in the elderly population (Shrock et al., 2020).

Senescent T cells from both mice and humans lose CD28 expression while gaining CD57 and KLRG-1 expression, similar to inflationary T cells (Akbar et al., 2016). T cell exhaustion, which occurs as a result of persistent viral infections or malignancies, is also more common among the elderly. T cells from the elderly specifically expressed higher levels of CTLA-4 (cytotoxic T-lymphocyte-associated antigen-4), PD-1 (programmed death-1), and TIM-3 (T cell immunoglobulin and mucin domain-containing protein 3) (Leng et al., 2002; Shimada et al., 2009; Lee et al., 2016). CTLA-4 and PD-1 are negative co-stimulatory/inhibitory receptors of T cells and both have been reported to be upregulated in the aged humans and mice T cells (Lee et al., 2016). TIM-3, an exhaustion marker of T cells, together with other inhibitory receptor PD-1 mediates CD8^+^ T cells exhaustion in terms of proliferative potential and cytokine production such as IL-2, IFN-γ, and TNF-α (Lee et al., 2016). The severity of COVID-19 is linked to lymphopenia, a condition characterized by remarkably low numbers of lymphocytes. The link between poor prognosis and a decrease in the total number of peripheral T lymphocytes in the blood has been reported (Huang and Pranata, 2020). Immunosenescence contributes to weaker T cell responses, which might be worsened by a drop in T cell counts, and making lymphopenia shown in severe COVID-19 even more dangerous for the aged individuals. A cohort study of 522 COVID-19 patients found a substantial link between lymphopenia and age, with patients >60 years having a minimum number of total T cells in their blood (Diao et al., 2020).

## Tissue-Specific Immunity

### Bone Marrow

All hematopoietic cells are produced in bone marrow, which also serves as the site of B cell maturation where precursor cells acquire surface immunoglobulin (Zhao et al., 2012). Antigen-experienced adaptive immune cells thrive in the bone marrow (BM), which provides a favourable environment for their long-term survival. Effector/memory T cells and plasma cell precursors move to the BM after coming into contact with antigens, where they may live in survival niches without being exposed to antigens (Naismith et al., 2019). In addition, adaptive immune cells phenotype varies with age, as do BM niches, resulting in poor long-term immunological memory maintenance in the elderly. This process appears to be influenced by oxidative stress, age-related inflammation (inflammaging), and cellular senescence (Naismith et al., 2019). In the human BM, the impact of aging on the synthesis of certain survival factors for effector/memory T cells and long-lived plasma cells has been reported (Pangrazzi et al., 2017). Particularly, the levels of IL-7, a cytokine that promotes memory T cell survival, were decreased in BM in old age. According to Stephan *et al*, BM stromal cells taken from elderly donors expressed less IL-7 than stromal elements derived from young BM donors (Stephan et al., 1998). In the BM, IL-15 was shown to be elevated in old age, mainly favouring the maintenance of highly differentiated, senescent-like T cells (Herndler-Brandstetter et al., 2011; Pangrazzi et al., 2017). Furthermore, with aging in the BM, the plasma cell survival factor april (a proliferation-inducing ligand) was reduced whereas the IL-6 level was elevated. The chemokine CXCL-12, which aids in the recruitment of plasma cells into niches was unaffected by age (Hargreaves et al., 2001). As a result, while aging may affect adaptive immune cell survival in the BM, the migration of these cells to niches may not be compromised. In humans and mice, IL-15 accumulation in the BM is thought to be advantageous because it promotes the activation and proliferation of effector/memory CD8^+^ T cells. (Becker et al., 2005; Herndler-Brandstetter et al., 2011). Despite this, it is crucial to keep in mind that both IL-15 and IL-6 are not only involved in the maintenance of adaptive immune cells, but they are also pro-inflammatory cytokines, hence, their levels should be maintained. It has been hypothesized that inflammation may be accountable, or at least contribute, to age-related deficiencies in the maintenance of immunological memory in the elderly, and because these molecules were over-expressed in the aged BM (Naismith et al., 2019). As the frequency of CD8^+^CD28^–^ T cells in the BM increased with age, it has been observed that IL-15 may play a key role in the survival of this fraction of highly differentiated T cells (Pangrazzi et al., 2017). Furthermore, CD8^+^CD28^–^ T cells in the BM expressed a significant amount of IFN-γ and TNF; thus, BM niches essential for the long-term maintenance of memory T cells seem to become pro-inflammatory in old age, with CD8^+^CD28^–^ T cells supporting this process. Indeed, CD8^+^CD28^–^ T cells produce IFN-γ and TNF after being recruited to the BM, which may operate on BM cells to stimulate the production of IL-15 and IL-6, hence encouraging the attraction of new CD8^+^CD28^–^ T cells. As a result, it is believed that in the aged BM, a vicious cycle of inflammation may occur, and compromising the maintenance of immunological memory (Naismith et al., 2019). In addition, a reduction in cellularity in the bone marrow is the most obvious alteration with age. Adipocytes, which are found in bone marrow, are beneficial for the development of young individuals and also provide a protective role (Ma and Fang, 2013). Hematopoietic tissue takes up 40–60% of marrow space in young individuals, 20–40% in elderly persons, whereas adipocytes take up the rest. By the time a person reaches the age of 30, these adipocytes have nearly completely replaced the bone marrow (Veldhuis-Vlug and Rosen, 2018). When bone marrow cells were irradiated in tissue with a higher proportion of marrow adipose tissue, the replenishment of blood cells was hindered (Ambrosi et al., 2017) as the study has indicated a link between marrow adipose tissue and hematopoiesis impairment. It’s conceivable that the negative link between marrow adipose tissue and blood cell growth contributes to the elderly’s weakened immune response.

**TABLE 1:** Age related changes in the immune system are summarized below.

Cells	Immune response	Aging associated impairment
Monocytes/macrophages	Innate	Less phagocytic activity
		Downregulated MHC II expression
		Decreased ROS and cytokine production
		Reduced TLR expression (except for TLR5)
Dendritic cells	Innate	Decreased maturation and Ag presentation
		Reduced TLR expression and signaling
		Impaired Ag uptake
		Reduced CD80 and CD86 expression
Neutrophils	Innate	Reduced chemotaxis
		Downregulated MHC II expression
		Decreased ROS and cytokine production
		Altered TLR expression
		Decreased NET formation
NK cells	Innate	Decreased perforin degranulation
		Reduced cytotoxicity
B cells	Adaptive	Limited diversity in B cell receptor (BCR) repertoire
		Decreased numbers of naive or circulating B cells
		Reduced antibody affinity
		Diminished memory B cells homeostasis
T cells	Adaptive	Limited diversity in Tcell receptor (TCR) repertoire
		Relative decrease in naive T cells
		Relative increase in memory T cells
		Increased number of senescent T cells
		Increase of CD8 T_EMRA_ cells
		Expansion of inflationary CD8 T^+^ cells caused by chronic viral infections
		Reduced effector T cell response to novel Ag

### Thymus

The thymus is the principal organ for the maturation of T cells with a wide range of antigen-recognition capabilities. T cells mature in the thymus after originating in the bone marrow (Thapa and Farber, 2019). T cells proliferate in the thymus and differentiate into helper T cells, regulatory T cells, or cytotoxic T cells, and as well as memory T cells. They are subsequently delivered to peripheral tissues or circulated in the bloodstream or lymphatic system (Thapa and Farber, 2019). The thymus starts to shrink around puberty, a condition known as thymic involution that is evolutionarily and conservatively maintained in vertebrates (Gui et al., 2012). The capacity of an individual to fight novel pathogens is reduced as a result of this condition. Thymic involution is characterized by a gradual reduction in thymus size as well as diminished thymic structure as people become older. Thymopoiesis is reduced when the number of thymic compartments decreases (Gui et al., 2012). There are several plausible explanations which shows that why this thymic involution process takes place with age. First, since it produces huge numbers of cells, the thymus is highly metabolically active, and the body has a tendency to downregulate metabolic processes in order to preserve energy, as one gets older (Gui et al., 2012). Second, this is the age when a person has most certainly come into contact with the vast number of pathogens in their local surroundings. As a result, it is physiologically advantageous to reduce energy consumption on the creation of new T cells instead depending on memory cells that have the ability to sense previously experienced pathogens (Gui et al., 2012). Thymic involution could be one reason why the older have worse outcomes when they get infected with new infections. For combating viruses, CD8 T cells and CD4 Th1 cells are especially important. Because SARS-CoV-2 employs several methods to circumvent the innate immune response (De Wilde et al., 2017), such as masking PAMPs and inhibiting IFN signaling, the adaptive immune response becomes even more crucial. If the new T cell receptors (TCR) are not produced, the individual will be unable to generate an effective immune response against any novel pathogens.

### Spleens

The spleen performs an essential function in fighting off infections in the bloodstream and triggering an adequate immune response to them (Bronte and Pittet, 2013). Usually, B cells in the marginal zone (MZ) of the spleen grab antigen and immune complexes from the blood and shuttle them to follicular dendritic cells in B-cell follicles, subsequently conducting reaction in the germinal center to T-independent antigens (Cerutti et al., 2013). It has been studied that antigen capture by MZ B cells in the spleens of elderly mice was decreased, and so their tendency to move between the MZ and the follicles (Turner and Mabbott, 2017a). This impairment in the movement between the two locations in elderly mice’s spleen is due to the heightened response to the chemo-attractant sphingosine-1-phosphate (S1P), which tends to keep them in the marginal zone (Turner and Mabbott, 2017b). This may not be significant to the aged response to SARS-CoV-2, since it is a T-dependent antigen, although it is an important factor to be considered (Bajaj et al., 2021).

### Lymph Nodes

Lymph nodes are secondary lymphoid organs that are found throughout the lymphatic system. They work to keep the organism healthy by filtering harmful particles out of the lymphatic fluid (Turner and Mabbott, 2017a). Antigen recognition, B and T cell activation, and proliferation are all key processes that occur in lymph nodes. Lymph nodes in aged mice and humans exhibit reduced interactions between B and T cells, lower numbers of T cells, abnormal lymphocyte migration, and a reduction in the number and size of germinal centers (Turner and Mabbott, 2017b). These alterations have a detrimental influence on the establishment of an adaptive immune response, in addition to the loss of follicular areas and germinal centers. A variety of age-related changes are likely to blame for the loss of follicular structure. First, there is a deterioration of follicular dendritic cell network (FDCs) with age. These FDCs play a critical role in the maintenance of the reaction occurring in the germinal centers (Bajaj et al., 2021). Immune complexes are kept by FDCs for presentation to B cells leading to stimulate proliferation in the germinal center and isotype switching in order to establish an adequate immune response. In addition, CXCL13 (selectively chemotactic for B cells) expression in the follicle is lower in the aged lymph nodes. CXCL13 is expressed by both follicular dendritic cells (Wang et al., 2011) and germinal centers follicular helper T cells (Tfh cells) (Rasheed et al., 2006) in the B-cell follicles. This chemokine is required, together with its receptor CXCR5, for the homing of naive B lymphocytes into a lymph node’s follicle, where they might recognize antigens and activated with T cells prior to clonal expansion in the germinal centers. The fibroblastic reticular cells especially play a critical role in the maintenance of naive T lymphocytes and lymph node development. Furthermore, a study revealed that the number of fibroblastic reticular cells and lymphoid endothelial cells had reduced in aged lymph nodes (Thompson et al., 2019). This study revealed that the outer capsule of aged lymph nodes in mice and rhesus macaques was thickened, the stroma was infiltrated with collagen, and the total size of the lymph node was reduced. Thus, these microscopic alterations may explain why the elderly lymph node is unable to maintain naive T cells that have recently moved into it (Thompson et al., 2019). For the novel T-dependent antigens like SARS-CoV-2, the retention and migration of naive T cells into the follicle is required for B cells activation, proliferation, and the appropriate immune response for the elimination of the virus (Bajaj et al., 2021).

## Comorbidities, Aging, and Disease Severity in COVID-19

The severity of COVID-19 disease is linked with numerous comorbidities and patients with old age (>60 years) (Callender et al., 2020). It has been speculated that when infected with SARS-CoV-2, individuals with comorbidities experience more serious clinical outcomes when compared to patients with no pre-existing comorbidities (Wang et al., 2020). The most common pre-existing comorbidity in COVID-19 patients has been reported as hypertension (Yang J et al., 2020; Zuin et al., 2020). According to a retrospective study, people with hypertension are at a higher risk of serious infection and death (Zuin et al., 2020; Lippi et al., 2020). Cardiovascular disease has also been found to be associated with several worse clinical outcomes with the elevated death rates in COVID-19 patients (Guan et al., 2020). COVID-19 severity was also shown to be higher in patients with type 2 diabetes, as it is the third most prevalent underlying symptomatology in COVID-19 patients (Zhu et al., 2020; Fadini et al., 2020). Diabetics are more susceptible to infection, and they are more likely to have several comorbidities, such as cardio-vascular disease (Guo et al., 2020). According to a recent report on COVID-19, diabetics were more susceptible to acquire pneumonia, accounting for 11.7% of serious cases but only 4% of mild/moderate cases (Li et al., 2020a) Obesity has also been linked to the majority of COVID-19 comorbidities, including hypertension, diabetes, and cardiovascular diseases (Balakumar et al., 2016; Kassir, 2020). Obesity prevalence varies widely over the world; for example, obesity is more prevalent in the United States and Europe than in Asian nations (Dietz and Santos-Burgoa, 2020). As a result, the severity of COVID-19 and death rates may differ. When comparing survivors versus non-survivors, research revealed that 88.2% of non-survivors had a BMI above 25 kg/m^2^, which was a much larger proportion than survivors (Liu M et al., 2020). Additionally, ACE2 expression was found to be elevated in the adipocytes of obese people, suggesting that it might be a potential target for SARS-CoV-2 infection (Kruglikov and Scherer, 2020). Chronic obstructive pulmonary disease (COPD), among several other comorbidities, and has been linked to poor disease progression. In patients with underlying COPD who were confirmed with COVID-19, a meta-analysis of numerous studies in China indicated a four-fold increase in death (Zhao et al., 2020). As per another meta-analysis study of 1786 COVID-19 patients, hypertension (15.8%), cardiovascular and cerebrovascular disorders (11.7%), and diabetes (9.4%) were reported as the most prevalent comorbidities (Paudel, 2020; Zhou F et al., 2020). While co-existing HIV and hepatitis B infection (1.5%), malignancy (1.5%), respiratory diseases (1.4%), renal diseases (0.8%), and immunodeficiencies (0.01%) were reported as the least prevalent comorbidities (Paudel, 2020).

The elderly COVID-19 patients with chronic comorbidities like diabetes, hypertension, cardiovascular, and lung diseases are at a greater risk of acquiring the serious forms of the disease that usually gets fatal (Sanyaolu et al., 2020). For people above the age of 65 in the United States, cardiac disease is the leading cause of mortality, followed by chronic low respiratory disease in third place and diabetes mellitus in sixth place (Heron, 2019).

The adipokine enzyme DPP4 has also been considered as a crucial factor that can be correlated with worse outcomes post SARS-CoV-2 infliction (Bassendine et al., 2020). MERS-CoV employs this enzyme for entry into the target cells, while SARS-CoV-2 also binds to this enzyme through the S1 domain of the spike proteins (Li et al., 2020b). COVID-19 patients experience dysregulation in DPP4 functioning. DPP4 enzyme is present in salivary glands, liver, kidney, colon epithelial cells, alveolar cells type2, and capillaries as well as in immune cells such as T cells, B cells, and NK cells (Bassendine et al., 2020). The elevated expression of the DPP4 enzyme has been found in the elderly and also in many patients with diabetes or other metabolic disorders. Furthermore, it has been observed that adipose tissue is a source of DPP4 in mice models of diet-induced obesity (Bassendine et al., 2020). The broad expression of DPP4 might explain the vast range of COVID-19 symptoms. The related research, taken together, suggests a possible explanation for the link between pre-existing diseases, comorbidities, and poor clinical outcomes (Bassendine et al., 2020). DPP-4 inhibitors, widely used to treat type 2 diabetes mellitus, may be beneficial to COVID-19 patients with type 2 diabetes, as they can benefit from anti-inflammatory, anti-proliferative, and anti-fibrotic effects, in addition to glycemic regulation (Smelcerovic et al., 2020). Furthermore, DPP4 inhibitors are effective in the prevention and treatment of pulmonary fibrosis, heart disease, and kidney damage, and since these conditions are a long-term consequence of COVID-19, it is plausible to anticipate that DPP-4 inhibitors will be effective in reducing COVID-19’s long-term repercussions (Smelcerovic et al., 2020).

## Therapeutics

COVID-19 has been linked to an increase in mortality in aged people, which has a great deal of correlation to immunosenescence, comorbidities, and the accompanying proinflammatory condition that develops as people age (Franceschi et al., 2000; Shaw et al., 2013; Weyand and Goronzy, 2016; Fuentes et al., 2017; Fulop et al., 2018). Senolytics, commonly known as “anti-aging medications,” eliminate senescent immune cells that build up over time and overproduce cytokines (Sargiacomo et al., 2020). Senolytics diminish the cytokine storm induced by excessive production of proinflammatory cytokines, may be beneficial to older persons. Drugs with senolytic properties, such as rapamycin and azithromycin, were widely employed as potential therapies for COVID-19, and it may help older persons in particular (Ma and Fang, 2013; Rea et al., 2018; Omarjee et al., 2020; Sargiacomo et al., 2020). Other medications that limit cytokine signaling or reduce inflammation, such as JAK-STAT pathway inhibitors and corticosteroids, may also help to reduce inflammation, particularly in aged COVID-19 patients (Rea et al., 2018; Gozzetti et al., 2020; Jamilloux et al., 2020; Sargiacomo et al., 2020).

### Senolytics

Several clinical trials are being conducted to investigate the impact of senolytic drugs in the aging process (Kirkland and Tchkonia, 2020; Bartleson et al., 2021). Senolytics like fisetin, dasatinib, and quercitin (D + Q), remove senescent cells and diminish the SASP’s pro-inflammatory and pro-thrombotic actions (Bartleson et al., 2021). Senolytics have been shown to relieve inflammatory symptoms in a few clinical trials. For instance, during idiopathic pulmonary fibrosis which is an age-related lung disease (Justice et al., 2019), D + Q lowered SASP-associated pro-inflammatory cytokines, and a current finding found that fisetin and D + Q lowered SASP and enhanced survivability in a mouse model of β-coronavirus (Camell et al., 2021).

### IL-6 Inhibitors

COVID-19 patients exhibit a greater level of serum IL-6, which has been linked to a more severe illness and death. As a result, IL-6 dysregulation might cause problems in recovery. As part of the inflammaging phenotype, older persons have been found to have higher serum IL-6 levels, predisposing them to a more severe COVID-19 manifestation (Rea et al., 2018). The Chinese Health Commission and the Italian Society for Infectious and Tropical Diseases both had suggested the use of anti-IL-6 monoclonal antibodies (mAbs) such as tocilizumab in COVID-19 treatment. Anti-IL-6 mAbs have also been shown to enhance clinical outcomes in COVID-19 patients (Jamilloux et al., 2020). Siltuximab, like tocilizumab, is an anti-IL-6 mAb with a greater affinity for IL-6. Antibiotics like azithromycin, in addition to mAbs, can also be used to block IL-6. In addition to suppressing IL-1 and IL-6 cytokines, azithromycin increases the destruction of senescent cells (Sargiacomo et al., 2020).

### Chloroquine/Hydroxychloroquine

The antimalarial, chloroquine (CQ) and its less toxic analog, Hydroxychloroquine (HCQ), have been demonstrated to affect the glycosylation of ACE2 receptor, hence disrupting the viral entry into the host cell (Liu et al., 2020). Furthermore, HCQ has been demonstrated to have anti-inflammatory properties, implying that it may aid in the reduction of pro-inflammatory cytokines release. However, clinical investigations employed with CQ/HCQ treatment have shown unsatisfactory outcomes that imply more danger than benefit. It has been suggested that HCQ and azithromycin, a macrolide antibiotic, may function together to reduce viral load *in vivo* and *in vitro* cell cultures (Andreani et al., 2020). These medications, however, are not without danger. The drugs such as HCQ, CQ, and azithromycin may cause cardiac arrhythmias by prolonging the QT interval (Kamp et al., 2020). Several COVID-19 patients have died as a result of severe cardiac events, presumably because of the overdosing of the above-specified drugs (Kamp et al., 2020). Surprisingly, in randomized controlled studies, HCQ was proven to be ineffective as a COVID-19 prophylactic or therapy (Boulware et al., 2020; Linsell and Bell, 2020). More clinical trials are required to explore the role of HCQ/CQ in COVID-19 patients.

### Corticosteroids

Corticosteroids are anti-inflammatory agents. Glucocorticoids were frequently used to treat systemic inflammation in SARS patients during the 2003 outbreak, suggesting that they may be used to treat COVID-19 individuals with identical symptoms (Zhang W. et al., 2020). However, using corticosteroids in the treatment of COVID-19 involves risks such as reduced viral clearance and an increased risk of subsequent bacterial or fungal infections (Russell et al., 2020). Due to inconsistent data and the possibility of bias, more information is needed to fully understand the function of corticosteroid usage in COVID-19 patients.

### JAK-STAT Pathway Inhibitors

Ruxolitinib and baricitinib are Janus kinase (JAK) inhibitors, have been discovered as potential COVID-19 treatment drugs. Despite the fact that JAK inhibitors may lower cytokine storms and alleviate COVID-19-induced ARDS, existing research is restricted by small sample sizes and inadequate experimental design (Bajaj et al., 2021).

### Remdesivir

Remdesivir, a nucleoside analog that blocks viral replication in SARS-CoV and MERS-CoV, has been discovered as a potential COVID-19 therapeutic option. Remdesivir may significantly minimize recovery time, according to preliminary data from a double-blind, randomized, and controlled experiment (Beigel et al., 2020). Remdesivir was linked to a reduced risk of serious side effects, such as respiratory failure. Provisional data supports the use of remdesivir to cure COVID-19 patients (Beigel et al., 2020). However, new information from the WHO Solidarity experiment, a randomized controlled trial with a huge enrollment of over 11,000 individuals, contradicts remdesivir’s success (Consortium et al., 2020). To explain the contradictory conclusions of the study, more evidence is required.

### Dipeptidyl Peptidase-4 -Inhibitor

Dipeptidyl peptidase-4 (DPP-4) inhibitor has anti-inflammatory, anti-fibrotic, and anti-adipogenic characteristics, which could be effective in inhibiting the progression to hyper-inflammation in extreme cases of COVID-19 (Strollo and Pozzilli, 2020; Iacobellis et al., 2020a; Valencia et al., 2020). Some adverse effects may be associated with using DPP4 inhibitors such as gastrointestinal complications, upper respiratory tract infection, headache, and allergic reactions (Pathak and Bridgeman, 2010). However, it has been observed that the usage of DPP-4 inhibitors was linked to decreased mortality in COVID-19 patients (Rakhmat et al., 2021). The use of DPP4 inhibitors, such as gliptins, in COVID-19 patients with or without type 2 diabetes may provide a convenient strategy to minimize viral entrance and reproduction into the airways, as well as to lessen the long-term cytokine storm and inflammation in the lungs has been studied (Solerte et al., 2020).

## Conclusion and Future Perspectives

The COVID-19 pandemic has exhibited a significant demographic bias, in terms of the number of cases and associated fatalities, where the elderly subjects succumb to the infection more predominantly. Apart from genetics and basic comorbidities, aging invokes a variety of physiological alterations in the immune system including immunosenescence and inflammaging, *etc*. As a consequence, the ability of the aged host immune system to combat invading pathogens deteriorates to a great extent. In fact, besides more predispositions to various infections, the elderly subjects generally fail to manifest desirable immune responses against vaccines and other prophylactic programs as well. The aging process modulates the proinflammatory phenotypes, which not only influence the vulnerability of an individual to various invading pathogens, but also to the course of the disease and clinical consequences. Recently, immunosenescence has been correlated with mortality among the elders with COVID-19. It has been suggested that inadequate T cell response, poor antibody generation against SARS-CoV-2, and inflammaging, etc., that severely destroys the homeostasis, ensues in severe organ failure of the aged COVID-19 patients. In order to customize therapies and vaccine approaches in the direction of personalized medicine, a better knowledge of the intricacies related to the immune system of elderly people, is required. The pathogenesis of COVID-19 is tremendously complicated, involving the inhibition of the host’s antiviral and innate immune responses, the promotion of oxidative stress, and hyper-inflammation characterized as a “cytokine storm,” resulting in acute lung damage, tissue fibrosis, and pneumonia, *etc*. (Mrityunjaya et al., 2020). Several strategies such as vaccines, nutraceuticals, and pharmaceuticals are currently being used to boost both immunity of the susceptible host on one hand as well as inhibition of the replication and vital cellular machinery of the SARS-CoV-2 on the other. There are some approved vaccines, such as Pfizer, Moderna, AstraZeneca, Johnson and Johnson, and Gam-COVID-vac (Sputnik V) (Teijaro and Farber, 2021), that trigger innate and adaptive immune responses against the deadly SARS-CoV-2 virus. Phase III clinical trials have demonstrated 90–95% efficacy of Pfizer/BioNTech (BNT162b2) and Moderna (mRNA-1273) mRNA vaccines, while the AdV vaccines (ChAdOx1nCoV-19), and Gam-COVID-vac (Sputnik V) showed slightly lower efficacy (average 70 and 91%, respectively) (Teijaro and Farber, 2021). However, their effectiveness in elderly subjects is still an enigma and needs time to establish their usefulness. On the other hand, nutraceuticals if used as an immune-boosting, antiviral, antioxidant, and anti-inflammatory agent can be more apposite in imparting desirable combat strategy against SARS-CoV-2 in general, as well as in elderly patients. It is speculated that nutraceuticals such as Zinc (Zn), vitamin D, vitamin C, cinnamaldehyde, curcumin, selenium, probiotics, quercetin, and lactoferrin, etc may help in fighting against COVID-19 (Mrityunjaya et al., 2020). Combining some of these phytonutrients in the proper mix as a nutritional supplement would also aid to boost the immune system, inhibit viral transmission, reduce disease development to the extreme level, and control excessive inflammation, thereby offering both prophylactic and therapeutic assistance against SARS-CoV-2 infection (Mrityunjaya et al., 2020). Some pharmaceutical candidates have been in various phases of research and development, while a few such as favipiravir, remdesivir, and tocilizumab, etc. have been repurposed and authorized for emergent use against the COVID-19 pandemic (Mrityunjaya et al., 2020).

It is critical to learn about the functioning of environmental factors like nutrition, physical activity, co-morbidities, and pharmaceutical therapies, and to the fact that how these modulate the aging immune system’s capacity to respond to invading pathogens on one hand and various vaccination programs on the other. The in-depth studies pertaining to COVID-19 have unraveled various relevant host immune system-related biomarkers as well as drug/antigen targets. More research is needed to figure out how to assist the elderly in generating a functional adaptive immune response while also reducing the detrimental pro-inflammatory state of the disease.
